# The fatty acid binding protein FABP7 is required for optimal oligodendrocyte differentiation during myelination but not during remyelination

**DOI:** 10.1002/glia.23789

**Published:** 2020-02-04

**Authors:** Sarah Foerster, Alerie Guzman de la Fuente, Yoshiteru Kagawa, Theresa Bartels, Yuji Owada, Robin J. M. Franklin

**Affiliations:** ^1^ Wellcome‐Medical Research Council Cambridge Stem Cell Institute, Jeffrey Cheah Biomedical Centre University of Cambridge Cambridge UK; ^2^ Department of Organ Anatomy Tohoku University Graduate School of Medicine Sendai Japan

**Keywords:** fatty acid binding protein, myelination, remyelination, OPC

## Abstract

The major constituents of the myelin sheath are lipids, which are made up of fatty acids (FAs). The hydrophilic environment inside the cells requires FAs to be bound to proteins, preventing their aggregation. Fatty acid binding proteins (FABPs) are one class of proteins known to bind FAs in a cell. Given the crucial role of FAs for myelin sheath formation we investigated the role of FABP7, the major isoform expressed in oligodendrocyte progenitor cells (OPCs), in developmental myelination and remyelination. Here, we show that the knockdown of *Fabp7* resulted in a reduction of OPC differentiation in vitro. Consistent with this result, a delay in developmental myelination was observed in *Fabp7* knockout animals. This delay was transient with full myelination being established before adulthood. FABP7 was dispensable for remyelination, as the knockout of *Fapb7* did not alter remyelination efficiency in a focal demyelination model. In summary, while FABP7 is important in OPC differentiation in vitro, its function is not crucial for myelination and remyelination in vivo.

## Graphical abstract

- FABP7 is required for OPC differentiation in vitro
- FABP7 is required for developmental myelination in vivo
- FABP7 is not required for remyelination in vivo

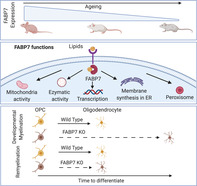

## 1 INTRODUCTION

The myelin sheath of the central nervous system (CNS) has a unique molecular composition that distinguishes it from other cell membranes: it comprises 71–81% of lipids, which include cholesterols (26%), galactolipids (31%), and phospholipids (44%) (Norton & Poduslo, 1973). The specific lipid composition allows close compaction of the myelin sheath, creating a highly ordered, hydrophobic barrier that enables myelin to function as an electric insulator (Simons & Nave, 2015).

Fatty acids (FAs) are the building blocks of the galactolipids and phospholipids found in myelin. To maintain water solubility and prevent aggregation, FAs are bound to proteins in the cytoplasm (Cistola, Hamilton, Jackson, & Small, 1988). There are ten FA binding proteins (FABPs), of which FABP3, FABP5, and FABP7 are expressed in the CNS (Owada, Yoshimoto, & Kondo, 1996). FABP7 is mainly expressed in astrocytes (Owada et al., 1996) and oligodendrocyte progenitor cells (OPCs; Sharifi et al., 2013), a progenitor cell giving rise to mature oligodendrocytes (OLs) in development and in response to injury (Franklin & ffrench‐Constant, 2017). OPCs isolated from *Fabp7* knockout (*Fabp7*KO) mice have impaired differentiation in vitro (Sharifi et al., 2013). In animal models of primary demyelination, *Fabp7* expression is increased in CNS resident cells (Bannerman, Hahn, Soulika, Gallo, & Pleasure, 2006; Huang et al., 2011; Kipp et al., 2011). However, the importance of FABP7 in the OPCs biology in vivo is not well understood.

In this study, we found that the pattern of FABP7 expression closely follows the timeline of myelination during postnatal development. This suggested a role in developmental myelination which was confirmed by a delay in developmental myelination in *Fabp7* knockout mice. From early adulthood onward, the expression of FABP7 was dramatically reduced and the protein was only re‐expressed following a demyelinating insult. However, *Fabp7*KO mice showed no effect on the remyelination capacity of OPCs.

## 2 MATERIALS AND METHODS

### 2.1 Animal husbandry

Animal experiments conformed to the UK Animals (Scientific Procedures) Act 1986 and were approved by the Cambridge University local ethical committees before licensing by the UK Home Office. The animals were housed under standard laboratory conditions on a 12 hr light/dark cycle with constant access to food and water. *Fabp7*KO mice were obtained from the Owada laboratory, Tohoku University, Japan (Owada et al., 2006). Demyelination experiments in the *Fabp7*KO animals were performed at Tohoku University, Japan. The experimental protocol for performing demyelinating lesions was reviewed by the ethics committee for Animal Experimentation of Tohoku University Graduate School of Medicine and carried out according to the guidelines for animal experimentation of the Tohoku University Graduate School of Medicine and under the law and notification requirements of the Japanese government.

### 2.2 Isolation of primary OPCs

OPCs were isolated from neonatal (P7–P30), 2 months, 3 months, 9 months, 12 months, or 24 months old Sprague Dawley rats as published previously (Neumann et al., 2019; Segel et al., 2019). Briefly, brains were digested in a papain solution (34U/ml, Worthington) containing DNAse Type IV (20μg/ml, Gibco) for 30–40 min at 37°C. The tissue was then triturated into a single cell suspension in Half (made *in house*) supplemented with B27 (1×, Gibco) and sodium pyruvate (2 mM, Gibco). After trituration, the single cell suspension was filtered through a 70 μm strainer and separated from debris by gradient density centrifugation (800 g, 20 min, RT) using 22.5% Percoll® (GE Healthcare). The cell pellet was then subjected to red blood cell lysis using the red blood cell lysis buffer (Sigma) for 2 min. Finally, OPCs were purified using the anti‐A2B5 microbeads MACS® cell separation system according to the manufacturer's protocol (Miltenyi Biotech). If used for Western blot analysis, obtained OPCs were resuspended in IP lysis/wash buffer (25 mM Tris‐HCl pH 7.4, 150 mM NaCl, 1 mM EDTA, 1% NP‐40, and 5% glycerol; Thermo‐Fisher Scientific). If used for qRT‐PCR analysis, obtained OPCs were resuspended in TRIzol Reagent (Thermo‐Fisher Scientific).

### 2.3 Isolation of OPCs using mixed glia culture

OPCs were obtained from P0–P3 old Sprague Dawley rats following the protocol of McCarthy and de Vellis (McCarthy & de Vellis, 1980). Briefly, the cortex was digested in a papain solution (34U/ml, Worthington) for 1 hr at 37°C. Digestion was stopped by adding DMEM (Gibco) supplemented with 10% FBS (Biosera) and the tissue was spun at 300 g for 5 min. The tissue was resuspended in DMEM with 10% FBS and 1% penicillin/streptomycin and cells from two brains were plated in a poly‐d‐lysine (Sigma) coated T75 flask. Mix glial cells were cultured at 37°C and 5% CO_2_ for 10 days with media changes every 3 days. After 10 days in vitro (DIV), the flasks were subjected to a shake off protocol to separate OPCs from the rest of the glial cells (McCarthy & de Vellis, 1980). OPCs were cultured in OPC medium (DMEM F12 (Gibco), 2 mM sodium pyruvate (Gibco), 60 μg *N*‐acetyl‐cysteine (Sigma‐Aldrich), 5 μg/ml insulin (Gibco), 21 mM d‐glucose (Sigma‐Aldrich), 50 μg/ml apo‐transferrin (Sigma‐Aldrich), 16.1 μg/ml putrescine (Sigma‐Aldrich), 40 ng/ml sodium–selenite (Sigma‐Aldrich), and 60 ng/ml progesterone (Sigma‐Aldrich) with daily addition of 10 ng/ml PDGF‐AA and 10ng/ml bFGF (PeproTech) at 37°C and 5% CO_2_. Cells were plated on poly‐d‐lysine coated coverslips at a density of 20,000 cells/13 mm coverslip. Each *n*‐number represents an OPC isolation from independent mix glial cell culture preparations on different days, each preparation performed from four individual P0–P3 old Sprague Dawley rats.

### 2.4 Fabp7 siRNA knockdown

OPCs isolated from mixed glial cell cultures were cultured for 2 DIV with growth factors, then the medium was changed to OPC medium without penicillin/streptomycin overnight. Cells were transfected with 50 nM FABP7 siRNA or equivalent non‐targeting control (GE Healthcare) using 1% Lipofectamine siRNAMAX (Invitrogen) diluted in Opti‐MEM (Gibco) according to the manufacturer's protocol. Six hours after transfection, the medium was replaced by OPC medium without growth factors, thyroxine and triiodothyronine. After 48 hr of transfection, cells were fixed with 4% (w/v) PFA for 10 min if used for immunocytochemistry staining, lysed in IP lysis/wash buffer (Thermo‐Fisher Scientific) if used for Western blot analysis or TRIzol Reagent (Thermo‐Fisher Scientific) if used for qPCR.

### 2.5 Immunocytochemistry

After siRNA treatment, cells were blocked with 5% normal donkey serum (NDS) (Sigma‐Aldrich) supplemented with 0.1% Triton X‐100 (Sigma‐Aldrich) in PBS for 1 hr at RT. Then cells were incubated for 1 hr at RT with primary antibodies (Mouse anti‐CNPase antibody, 1:500 (C5922, Sigma‐Aldrich); Rabbit anti‐Olig2 antibody, 1:500 (AB9610, Millipore); or rat anti‐MBP antibody, 1:500 (MCA4095, Serotec)) in 5% NDS with 0.1% Triton X‐100. After three washes, the cells were incubated with the appropriate Alexa Flour secondary antibodies (1:500, Life‐technologies) diluted in 5% NDS with 0.1% Triton X‐100 for 1 hr at RT. Nuclei were stained with Hoechst (2μg/ml, Sigma‐Aldrich) for 10 min at RT, before the coverslips were mounted using Fluoromount G (Southern Biotech).

### 2.6 Western blot

Protein lysates were mixed with NuPage loading buffer and NuPage reducing agent (DTT) (Life‐technologies) according to manufacturer's protocol and boiled for 10 min at 95°C. After denaturation, 5 μg of each sample were loaded on 4–12% Bolts Gels (Life Technologies) and the gels were subjected to electrophoresis at 120 V for 2 hr. In the sumoylation and ubiquitination experiments, 1 μg ubiquitin (U‐100H, R&D Systems) and Sumo 1, 2, and 3 (K700, R&D Systems) were loaded as control. The gels were then transferred onto a methanol preactivated PVDF membrane (Millipore) using a wet transfer tank (Bio‐Rad) and 1× NuPage transfer buffer (Life‐technologies) with 20% ethanol for 90 min at 100 V. The PVDF membrane was then blocked in blocking buffer (TBS blocking agent 1:1 (Li‐Cor) with TBS (Fischer scientific)‐0.1% Tween (Sigma‐Aldrich) or 5% skimmed milk in TBST (TBS with 0.1% Tween)) for 1 hr at RT. PVDF membranes were incubated overnight at 4°C with the primary antibodies (Rabbit anti‐BLBP, 1:500 (ab32423, Abcam); Mouse anti‐actin, 1:5000 (A5441, Sigma); Mouse anti‐actin (sc‐47778, SantaCruz Biotechnology); Mouse anti‐ubiquitin, 1:200 (sc8017, Santa Cruz); or mouse anti‐sumo1 antibody, 1:500 (sc5308, Santa Cruz). Subsequently the membranes were incubated with secondary antibodies (Donkey anti‐rabbit 680, 1:10,000 (926_38073, Li‐Cor); donkey anti‐mouse 800, 1:10,000 (926_32212, Li‐Cor); goat anti‐mouse IgG‐HRP conjugated (AP307P, Millipore) or goat anti‐mouse IgG‐HRP conjugated (AP124P, Millipore)). If appropriate, membranes were then incubated with an actin‐peroxidase antibody, 1:25,000 (A3854, Sigma) for 20 min at RT. Fluorescent and HRP signals were detected using the Odyssey apparatus (Li‐Cor) with an exposure time of 2 min.

### 2.7 Phosphorylation and glycosylation experiments

Cells were lysed in IP lysis/wash buffer (Thermo‐scientific) without the addition of protease and phosphatase inhibitors. For glycosylation analysis, 5 μg OPC lysates were then subjected to digestion for 1 hr at 37°C with EndoH enzyme or EndoH glycobuffer (NEB) alone as control. For phosphorylation analysis, 5 μg of OPC lysate were subjected to digestion for 30 min at 30°C with Lambda protein phosphatase or 10× NeBuffer Protein MetalloPhosphatases (PMP) and MnCl_2_ only as control. The OPC lysates were then boiled for 10 min at 95°C and further processed for Western Blot as described above.

### 2.8 Real‐time qPCR

RNA was isolated from cultured OPCs according to the RNAeasy Mini Kit (74104, Qiagen). All RNA samples were stored at −80°C prior to further processing. cDNA was generated using the QuantiTect Reverse Transcription Kit according to the instructions of the manufacturer (205310, Qiagen). For RT‐qPCR, *Fabp7* primers (forward (5′ ➔ 3′): AAGATGGTCGTGACTCTTAC; reverse (5′ ➔ 3′): GGAAACCAAGTTGTCAAAAG) were used at a concentration of 400 μM. The efficiency of the primer was greater than ~95% as determined by serial dilutions of OPC cDNA. cDNA, primers, and the SYBR Green Master Mix (204141, Qiagen) were mixed as instructed by the manufacturer, and RT‐qPCR and melting curve analysis were performed on Life Technologies' QuantStudio 6 Flex Real‐Time PCR System. Fold changes in gene expression were calculated using the ΔΔCt method in Microsoft Excel.

### 2.9 Toxin induced (lysolecithin) demyelination model in the spinal cord

For spinal cord lysolecithin lesions 2–3 months old wild type and homozygous *Fabp7* knockout mice (Owada et al., 2006) were used. Demyelination was induced in the caudal thoracic ventral funiculus of the spinal cord by injection of 1% (v/v) lysolecithin as previously described (Fancy et al., 2009).

### 2.10 Immunohistochemistry

Mice were terminally anaesthetized and fixed by intracardiac perfusion at 5, 10, or 21 days post lesion (dpl) induction using 4% (w/v) PFA. Spinal cords were removed, postfixed in 4% (w/v) PFA overnight at 4°C, cryoprotected with 20% (w/v) sucrose for 24–48 hr, embedded, frozen in OCT medium and stored at −80°C. Tissues were sectioned at 12 μm and collected onto poly‐l‐lysine‐coated glass slides. 12 μm cryo‐sections were dried at RT and then rehydrated in PBS. After rehydration, slides were postfixed for 10 min with 4% (w/v) PFA and then washed 3× 10 min with PBS. Sections were blocked with 5% NDS and 0.1% Triton X‐100 for 1 hr at RT. In case the mouse‐anti APC antibody was used, the slides were blocked using the MOM Kit according to the manufacturer's instructions (BMK‐2202, Vector Laboratories). After blocking, slides were incubated with primary antibodies in blocking solutions overnight at 4°C (Rabbit anti‐OLIG2, 1:500 (AB9610, Millipore); Goat anti‐SOX10, 1:100 (sc365692, Santa Cruz); Rabbit anti‐Ki67, 1:500 (ab16667, Abcam); or mouse anti‐APC, 1:300 (OP80, Millipore). Slides were then incubated with the appropriate Alexa Fluor® secondary antibodies 1:500 (Life Technologies) for 2 hr at RT. Nuclei were stained with Hoechst (2 μg/ml, Sigma‐Aldrich) for 10 min at RT, before the coverslips were mounted using Fluoromount G (Southern Biotech).

### 2.11 Toluidine blue staining

For toluidine blue and electron microscopy experiments, mice were terminally anaesthetized and fixed by intracardiac perfusion at 14 and 21 days post lesion (dpl) using 4% (w/v) glutaraldehyde. Spinal cords were removed and postfixed in 4% (w/v) glutaraldehyde overnight at 4°C. The tissue was dehydrated in a series of ethanol washes (1× 70% EtOH for 15 min, 1× 95% EtOH for 15 min, and 3× 100% EtOH for 10 min (Sigma)), washed twice in propylene oxide for 15 min and incubated in a one‐to‐one mix of propylene oxide and resin (50% resin, 34% dodecenyl succinic anhydride (DDSA), 16% methyl nadic anhydride (MNA), 2% 2,4,6‐Tris(dimethylaminomethyl)phenol (DMP‐30), all (v/v), TAAB Laboratories) for at least 3 hr at RT. The tissue was then incubated twice in pure resin for 12 hr at RT, before samples were embedded in plastic containers in fresh resin and hardened for 2 days at 60°C. Samples were cut into 0.75 μm resin sections and sections were then stained for 30 s with toluidine blue at 65°C on a heat plate. The toluidine blue images were blindly ranked by two independent assessors, who ranked them according to their level of demyelination and remyelination. In resin sections, remyelinated axons can be readily distinguished from normally myelinated axons outside the lesion by the thinness of the myelin sheath. Within the lesion, remyelinated axons can be distinguished from demyelinated axons because the former have myelin sheaths recognizable as a dark staining rim around the axon. The highest rank was given to the animal exhibiting the highest proportion of remyelinated axons. If it was not possible to differentiate two animals using this method then they were given the same rank. In this method, no attempt is made to assign a value to the proportion of remyelination, but simply to establish how a section from an individual animal ranks relative to others.

### 2.12 Quantification

To quantify in vitro proliferation and differentiation assays, five randomly chosen areas of the coverslip were imaged per condition with the 20× objective of the SP5 Leica confocal with a 512 × 512 resolution and 2 μm stacks. Counting was performed manually using the cell counter plugin in ImageJ (Version 2.0.0‐rc‐68/1.52 hr). To quantify remyelination efficiency, three lesions per animal were imaged with the 20× objective (SPR Leica Confocal) at a 512 × 512 resolution and 2 μm stacks. Using ImageJ software (Version 2.0.0‐rc‐68/1.52 hr), the lesion area was delineated and measured, and the number of different cell types within the lesion was counted manually with the cell counter plugin. To quantify OPC differentiation during developmental myelination, white matter of three sections per animal were imaged using the 20× objective (Leica SP5 confocal) at 512 × 512 resolution with 2 μm stacks. The area of interest was measured using Image the J software and the number of cells positive for the indicated marker proteins within the area of interest was counted manually. Toluidine blue staining was imaged using 63× objective of the Zeiss Apotome with a 2048 × 2048 resolution. White matter of three sections per animal were imaged and the number of myelinated axons in the white matter were manually counted using the cell counter plugin in ImageJ (Version 2.0.0‐rc‐68/1.52 hr). Western blots were quantified by measuring the integrated density of each of the bands by the quantification tool in the Image studio software (Version 4.0, Li‐Cor).

### 2.13 Statistics

Statistical analysis was performed using the Prism 7.0 and 8.0 (GraphPad Software) and SPSS Statistics 20.0 (IBM). Mean ± *SEM* are shown in all the graphs. The data were analyzed for normal distribution using D'Agostino‐Pearson omnibus and Shapiro–Wilk normality test. If the number of biological replicates was low (n < 4), normality was calculated using normality via residuals. A two‐tailed unpaired Student's *t*‐test was performed to assess the statistical significance between two groups. In case the data were not normally distributed, a *U*‐Mann–Whitney test was performed. When comparing more than two groups, a one‐way Anova test was used followed by a Tukey's posthoc test. If the sample was not normally distributed, a Kruskal–Wallis test combined with a Dunn's posthoc test was carried out. Remyelination ranking was evaluated using a *U*‐Mann–Whitney test. Western‐blot data were analyzed using a one‐way ANOVA and the corresponding Bonferroni posthoc test. qPCR data were analyzed using a one sample *t*‐test. In case the data were shown in percentages, adequate arcsin conversion was done prior to the unpaired Student's *t*‐test.

## 3 RESULTS

**Figure 1:**
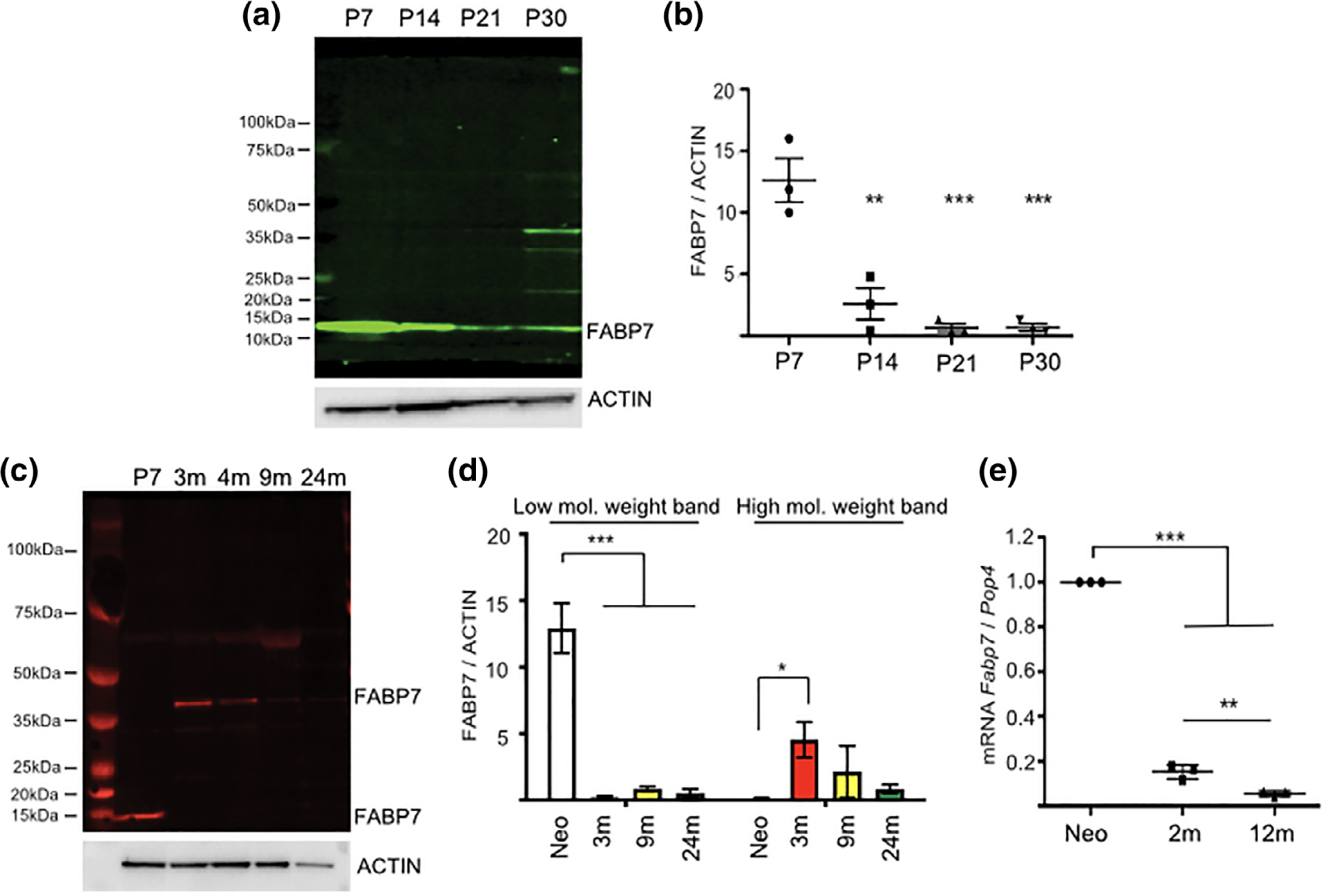
FABP7 expression in OPCs. (a) Western blot of FABP7 expression in acutely isolated OPCs at different developmental time points (P = postnatal day). (b) Quantification of FABP7 protein expression normalized to actin (one‐way ANOVA, *p* = .0001, Bonferroni post hoc test; (1) P7 versus P14, *p* = .0010; (2) P7 versus P21, *p* = .0003, (3) P7 versus P30, *p* = .0003; *n* = 3, mean ± *SEM*). (c) Western blot of FABP7 expression in ageing OPCs: note the higher molecular weight band appearing in adult OPCs. (d) Quantification of FAPB7 protein expression of the lower and higher molecular weight bands normalized to actin (low molecular weight band: one‐way ANOVA, *p* < .0001, Bonferroni posthoc test; Neo (P7) vs 3, 9, and 24 months, p<0.0001; High molecular weight band: one‐way ANOVA, *p* = .0366, Bonferroni posthoc test; Neo (P7) verssus 3 months, *p* = .0300; *n* = 3, mean ± *SEM*). (e) qPCR quantification of the relative expression of *Fabp7* mRNA in ageing OPCs normalized to *Pop4* (housekeeping gene) (one‐way ANOVA; *p* < .0001, Bonferroni post hoc test; (1) Neo (P7) versus 2 and 12 months, *p* < .0001; (2) 2 versus 12 months, *p* = .0030; *n* = 3, mean ± *SEM*)

FABP7 was highly expressed in OPCs isolated from rat brain at postnatal day 7 (P7; Figure 1a,b). However, with postnatal development, FABP7 protein expression steadily declined, reaching significance as early as P14 (P7 vs. P14, *p* = .001, Bonferroni posthoc test; Figure 1a,b). From P30, a higher molecular weight of FABP7 is detected at 40 kDa (Figure 1a,c). This is likely attributable to posttranslational changes as the adult form of FABP7 is more glycosylated and phosphorylated compared to its neonatal counterpart (Figure S1a). Ubiquitination and sumoylation do not contribute to the high molecular weight band in young adult OPCs (Figure S1b,c). With ageing, *Fabp7* mRNA and protein expression continue to decrease until it is undetectable in the aged rat (Figure 1c–e). The antibody used for the Western Blot analysis was specific to FABP7 protein as we did not detect FAPB7 protein in *Fabp7*KO animals, as it has been reported in a previous publication (Figure S1d; Driessen et al., 2018).

**Figure 2:**
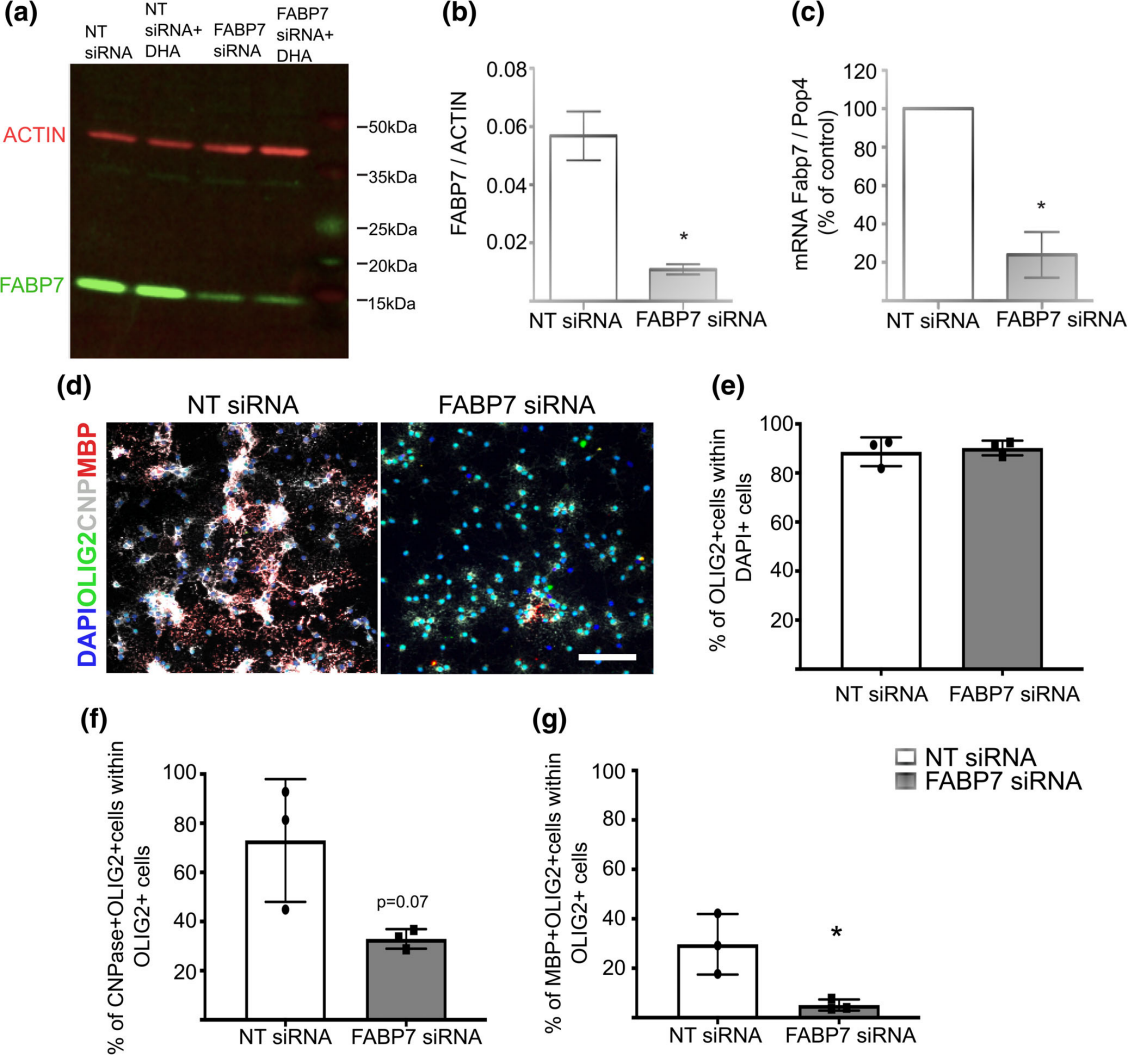
*Fabp7* siRNA knockdown affects OPC differentiation. (a) Western blot of FABP7 expression in OPCs in vitro after siRNA knockdown compared to scrambled siRNA control. NT = non‐targeting siRNA. DHA = docosahexaenoic acid. (b,c) Quantification of FABP7 protein (b) or mRNA (c) expression after siRNA treatment compared to scrambled siRNA control (unpaired Student's *t*‐test, *p* = .0135 (b); unpaired Student's *t*‐test, *p* = .0239 (c); *n* = 3, mean ± *SEM*). (d) Immunocytochemistry staining for OLIG2, CNPase, and MBP (marker of differentiated oligodendrocytes) in mixed glia derived OPCs after *Fabp7* siRNA knockdown. (Scale bar: 100 μm) (e–g) Quantification of the percentage of DAPI^+^ cells expressing OLIG2 (e), OLIG2^+^ cells expressing CNPase (f) or MBP (g) (unpaired Student's *t*‐test, *p* = .0701 (f), *p* = .0291 (g); *n* = 3, mean ± *SEM*)

As FABP7 is highly expressed in neonatal OPCs (Figure 1), we investigated its role in OPC proliferation and differentiation by knocking down *Fabp7* in vitro using siRNA. siRNA knockdown (KD) efficiency was >75% both on protein (Figure 2a,b) and RNA (Figure 2c) level. Mixed glial culture‐derived neonatal OPC cultures contained 90% Olig2^+^ cells (Figure 2d,e), minimizing any possible indirect effect on OPCs from *Fabp7*KD in other CNS cell types in vitro. Knockdown of *Fabp7* in these mixed glial culture‐derived neonatal OPCs resulted in a 2.2‐fold reduction of OPC differentiation into CNP^+^/OLIG2^+^ mature oligodendrocytes in differentiation medium from which the growth factors PDGF and FGF‐2 were removed (WT: 73% CNP^+^/OLIG2^+^ cells, *Fabp7*KD: 33% CNP^+^/OLIG2^+^ cells, *p* = .07, unpaired Student's *t*‐test; Figure 2d,f). Similarly, the proportion of OPCs differentiating into MBP^+^/OLIG2^+^ myelin‐sheath forming oligodendrocytes is also significantly reduced (WT: 27% MBP^+^/OLIG2^+^ cells, *Fabp7*KD: 5% MBP^+^/OLIG2^+^ cells, *p* = .03, unpaired Student's *t*‐test) (Figure 2d,g). These findings concur with a study by Sharifi and colleagues, whose data was also consistent with a role for FABP7 in OPC differentiation (Sharifi et al., 2013). In the same publication it was also shown that the knockout of *Fabp7* (*Fabp7*KO) affected the OPC proliferation capacity in vitro (Sharifi et al., 2013). In agreement with this study, we found that, while the number of SOX10^+^ cells stays constant (Figure S2a,b), *Fabp7*KD led to a reduction of the proportion of SOX10^+^ oligodendrocyte lineage cells that expressed KI67 (WT: 72% SOX10^+^/KI67^+^ cells, *Fabp7*KD: 61% SOX10^+^/KI67^+^ cells, *p* = .03, unpaired Student's *t*‐test; Figure S2a,c).

**Figure 3:**
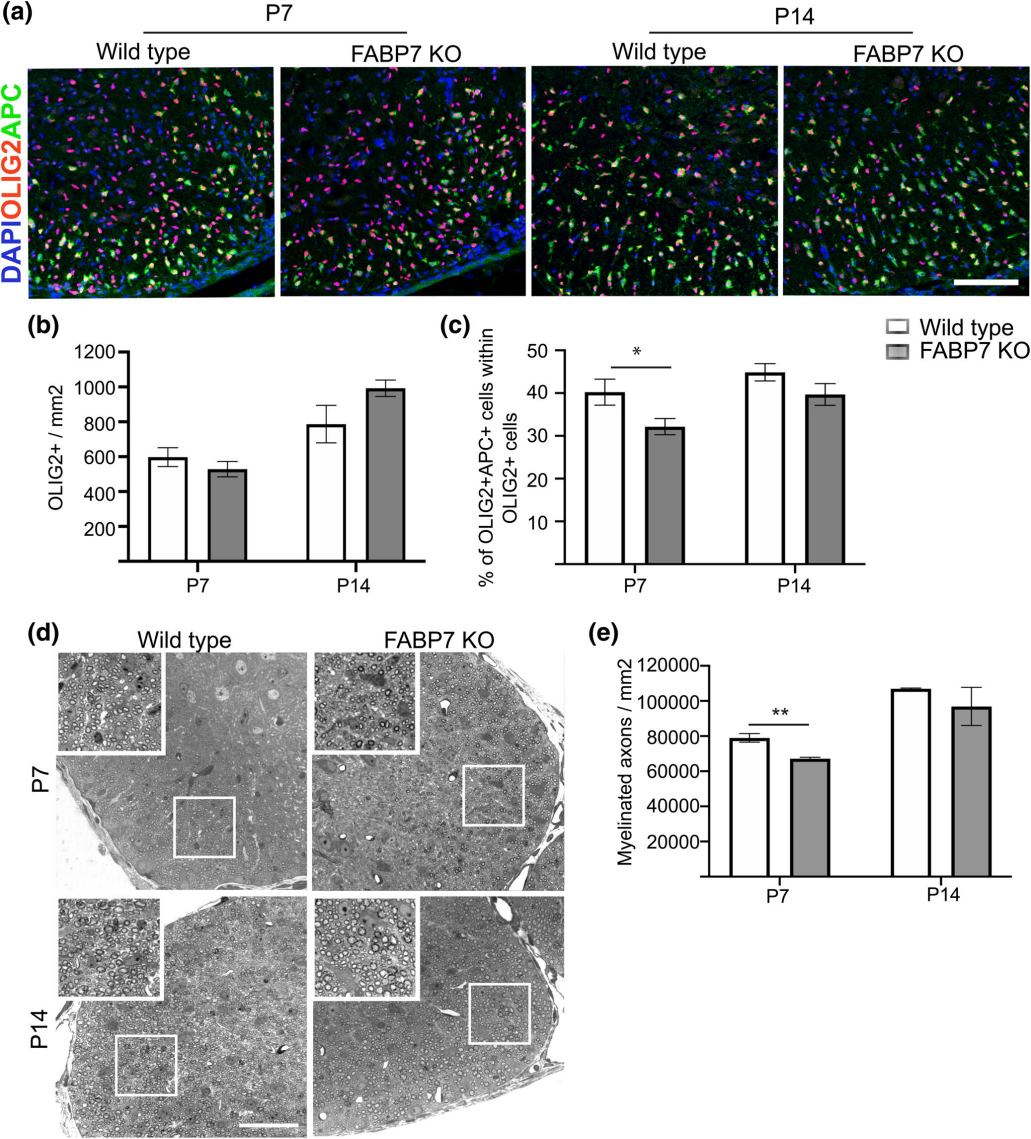
Developmental myelination in the spinal cord is delayed in *Fabp7* knockout mice. (a) Immunohistochemistry staining for OLIG2 and APC (marker of differentiated oligodendrocytes) in the ventral white matter of the spinal cord of WT and *Fabp7*KO mice at P7 and P14 (Scale bar: 100 μm). (b) Quantification of OLIG2^+^ cells per area in the whole spinal cord of WT and *Fabp7*KO mice at P7 and P14 (unpaired Student's *t*‐test, *p* = .3430 (P7) and *p* = .1089 (P14); *n* = 5–6, mean ± *SEM*). (c) Quantification of the percentage of OLIG2^+^APC^+^ in the white matter of the spinal cord of WT and *Fabp7*KO mice at P7 and P14 (unpaired Student's *t*‐test, *p* = .0435 (P7) and *p* = .1439(P14); *n* = 5–6, mean ± *SEM*). (d) Toluidine blue staining of the ventral spinal cord in WT and *Fabp7*KO mice at P7 and P14 (Scale bar: 50 μm). White squares highlight areas shown in the higher magnification insets. (e) Quantification of the number of myelinated axons per area in the ventral white matter at P7 and P14 (unpaired Student's *t*‐test, *p* = .009 (P7); *n* = 3 (P7), *n* = 2 (P14), mean ± *SEM*)

**Figure 4:**
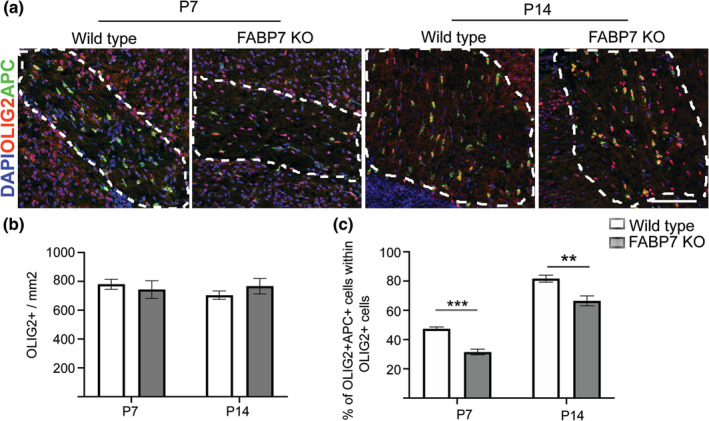
Developmental myelination in the brain is also delayed in *Fabp7* knockout mice. (a) Immunohistochemistry staining for OLIG2 and APC in the corpus callosum of WT and *Fabp7*KO mice at P7 and P14 (Scale bar: 100 μm). (b) Quantification of OLIG2^+^ cells per area in the corpus callosum of WT and *Fabp7*KO mice at P7 and P14 (unpaired Student's *t*‐test, *p* = .9307 (P7) and *p* = .3601 (P14); *n* = 4–5, mean ± *SEM*). (c) Quantification of the percentage of OLIG2^+^APC^+^ cells in the white matter of the corpus callosum of WT and *Fabp7*KO mice at P7 and P14 (unpaired Student's *t*‐test, *p* = .0001 (P7) and *p* = .0045 (P14); *n* = 4–5, mean ± *SEM*)

Given that the expression pattern of FABP7 is reminiscent of the timeline of developmental myelination (Figure 1) and FABP7 contributes to OPC differentiation in vitro (Figure 2), we investigated whether the knockout of *Fabp7* altered developmental myelination. The global homozygous knockout of *Fabp7* did not affect the density of oligodendrocyte lineage cells, identified by the expression of OLIG2 (Figure 3a,b). However, *Fabp7*KO mice showed a significantly reduced percentage of APC^+^/OLIG2^+^ mature oligodendrocytes in the spinal cord at P7 (WT: 40% APC^+^ OLIG2^+^, *Fabp7*KO: 32% APC^+^ OLIG2^+^, *p* = .04, unpaired Student's *t*‐test; Figure 3a,c). This corresponded with a decreased number of myelinated axons in the spinal cord at P7 (WT: 78977 myelinated axons/mm^2^, *Fabp7*KO: 67110 myelinated axons/mm^2^, *p* = .009, unpaired Student's *t*‐test; Figure 3d,e). However, this hypomyelination was transient, as the percentage of mature APC^+^/OLIG2^+^ oligodendrocytes, as well as the number of myelinated axons, was not significantly different in the spinal cord of WT or *Fabp7*KO mice at P14 (Figure 3a–e). As in the spinal cord, the percentage of APC^+^/OLIG2^+^ mature oligodendrocytes was significantly decreased in the corpus callosum of *Fabp7*KO animals at P7 (WT: 47% APC^+^/OLIG2^+^, *Fabp7*KO: 31% APC^+^/OLIG2^+^, *p* = .0001, unpaired Student's *t*‐test) and P14 (WT: 81% APC^+^/OLIG2^+^, *Fabp7*KO: 66% APC^+^/OLIG2^+^, *p* = .0045, unpaired Student's *t*‐test; Figure 4a,c), while no differences were detected in the density of OLIG2^+^ oligodendrocyte lineage cells (Figure 4a,b). The extended period of delayed myelination in the corpus callosum compared to the spinal cord is possibly due to a later onset of myelination in the corpus callosum. Unlike in the already published data (Sharifi et al., 2013) and our own in vitro results (Figure S2a,c), OPC proliferation was not altered by the absence of FABP7 in the spinal cord at P7 and P14 (Figure S2d,e). These data indicate that FABP7 plays a role in OPC differentiation, but not proliferation, during development.

**Figure 5:**
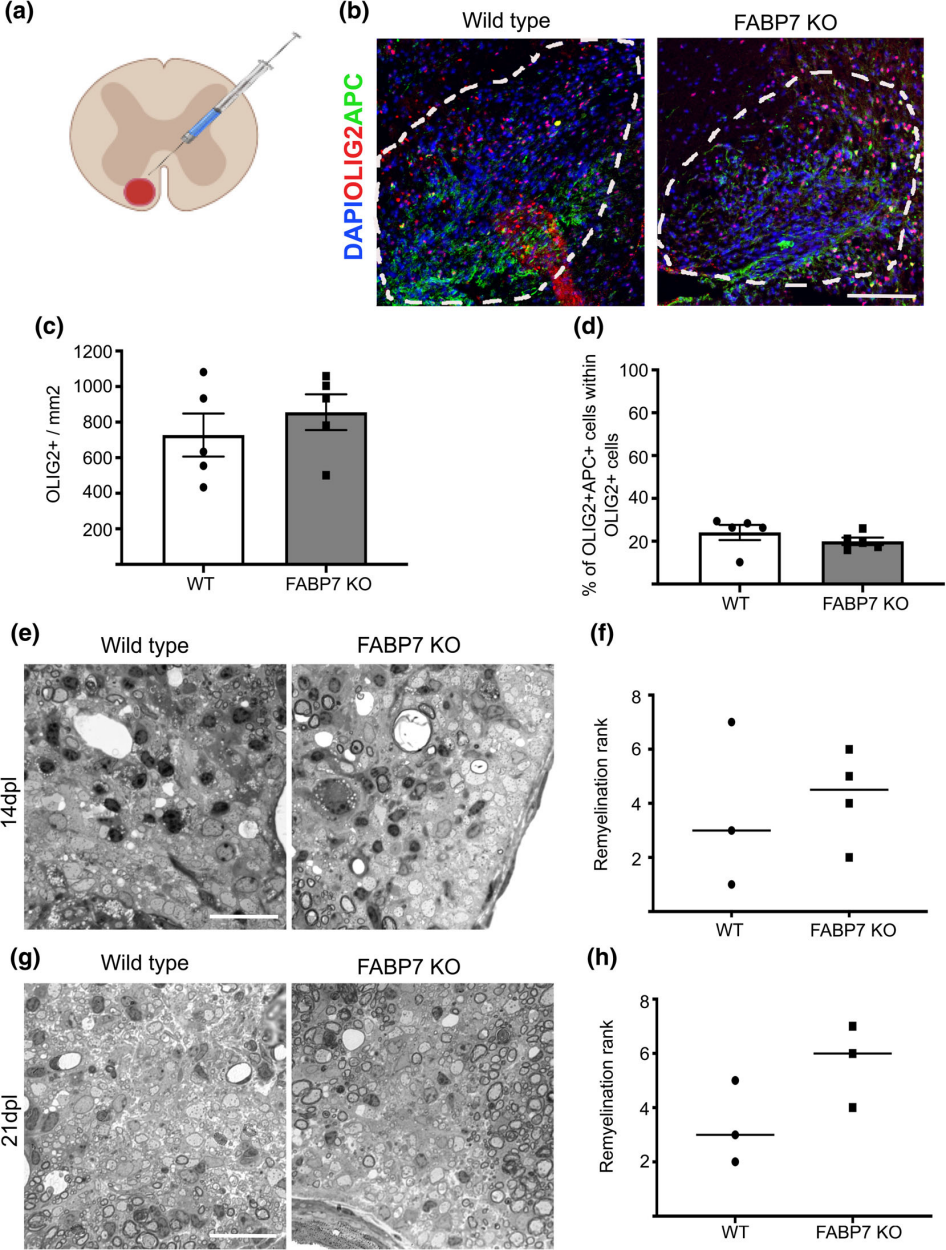
FABP7 is not essential for remyelination. (a) Schematic drawing of lysolecithin induced demyelination in the ventral white matter of the spinal cord. (b) Immunohistochemistry staining for OLIG2 and APC in the lesion in the ventral white matter of WT and *Fabp*7KO mice at 14 days post lesion (dpl) (Scale bar: 100 μm). (c) Quantification of OLIG2^+^ cells per area in the lesion of WT and *Fabp7*KO mice at 14dpl (unpaired Student's *t*‐test, *p* = .4371; *n* = 5, mean ± *SEM*) (d) Quantification of the percentage of OLIG2^+^APC^+^ cells in the lesion of WT and *Fabp7*KO mice at 14dpl (unpaired Student's *t*‐test, *p* = .3154; *n* = 5, mean ± *SEM*) (e,g) Toluidine blue staining of the lesion in WT and *Fabp7*KO mice at 14dpl (e) and 21dpl (g) (Scale bar: 25 μm). (f,h) Ranking analysis of remyelination efficiency in WT and *Fabp7*KO mice at 14dpl (f) and 21dpl (h) (*U*‐Mann–Whitney test, *p* = .8571 (f), *p* = .2000 (h); *n* = 3‐4, mean ± *SEM*)

OPCs generate oligodendrocytes not only during developmental myelination, but also for remyelination in response to demyelinating injury. Given the involvement of FABP7 in OPC differentiation during developmental myelination (Figures 3 and 4) and that FABP7 expression is increased at 14 days post lesion (dpl) during remyelination (Huang et al., 2011), we next asked whether FABP7 also plays a role in response to a toxin‐induced demyelination. To assess the remyelination capacity of *Fabp7*KO OPCs, we created a demyelinating lesion in the ventral spinal cord white matter of young adult mice by direct injection of lysolecithin (Figure 5a). There was no difference in the density of OLIG2^+^ oligodendrocyte lineage cells in the lesion (Figure 5b,c), neither in the percentage of APC^+^/OLIG2^+^ mature oligodendrocytes at 14 dpl (Figure 5b,d). Similarly, unbiased ranking of the proportion of remyelinated axons in the lesion did not show any significant difference between WT and *Fabp7*KO animals at 14 and 21 dpl (Figure 5e–h). Additionally, we also did not find a difference in the proliferation capacity of OPCs in WT and *Fabp7*KO after a demyelinating insult (Figure S3a–c), indicating that the loss of FABP7 in oligodendrocyte lineage cells does not impede their remyelination capacity.

## 4 DISCUSSION

### 4.1 FABP7 is involved in OPC differentiation in vitro

Here we confirm that FABP7 plays a role in OPCs differentiation in vitro (Figure 2), which agrees with an earlier study using a different experimental approach (Sharifi et al., 2013). The mechanism by which FABP7 modulates OPC differentiation are not known and require further exploration. FABP7 has high binding affinities to docosahexaenoic acid (DHA, 22:6(*n* − 3)), *α*‐linolenic acid (LA, 18:2(*n* − 6)), and eicosapentaenoic acid (EPA, 20:5(*n* − 3); Balendiran et al., 2000), thereby playing a central role in the intracellular transport of these FAs to various cellular organelles. In astrocytes, FABP7 can bind to PPAR‐*γ* (Tripathi et al., 2017) and modulate ERK phosphorylation (Yasumoto et al., 2018), both pathways involved in OPC maturation and differentiation (Fyffe‐Maricich, Karlo, Landreth, & Miller, 2011; Saluja, Granneman, & Skoff, 2001). However, whether the same pathways are employed in oligodendrocyte lineage cells to regulate their differentiation potential remains to be addressed.

### 4.2 FABP7 is dispensable for OPC differentiation in vivo

In *Fabp7*KO animals, developmental myelination was delayed at P7, but oligodendrocyte numbers recovered to physiological levels at P14 in the spinal cord (Figure 3). This delay in developmental myelination might be caused by a direct effect on the OPCs as suggested by the in vitro data (Figure 2). However, as the major FABP7 expressing cell type in the CNS are astrocytes it is also feasible that an indirect effect of the *Fabp7*KO in astrocytes attenuates OPC differentiation. Indeed, mice lacking connexin 47 and 30, preventing the coupling of astrocytes to oligodendrocytes, leads to a transient reduction in the number of oligodendrocytes and thinner myelin sheaths (Tress et al., 2012).

In the demyelination model, however, we did not observe a similar delay of OPC differentiation in *Fabp7*KO animals (Figure 5). While it is possible that a potential delay in OPCs differentiation in response to demyelination was not detected due to the time point chosen for analysis (14 and 21 days post lesion, Figure 5), there might also be differences in the FA transport between developmental myelination and remyelination. For example, changes in the lipid composition in the myelin sheath formed in remyelination have been reported (Wilson & Tocher, 1991), that might render FABP7 dispensable for remyelination.

Nevertheless, regardless of whether there is an undetected delay in OPC differentiation after demyelination, the absence of FABP7 does not have a long‐term effect on either developmental myelination or remyelination in vivo. A reason for its dispensability could be a compensatory mechanism in which other FABP isoforms, also physiologically expressed in the brain, are upregulated. However, no increase in FABP3/5 expression in response to *Fabp7* knockout has been observed in development and early adulthood (Owada et al., 2006), rendering the compensation by other FABP isoforms unlikely. As long chain FAs would aggregate in the cytoplasm, an alternative FA transport pathway must exist in oligodendrocytes. Elucidating these FA transport pathways in oligodendrocytes could provide new therapeutic targets to enhance OPC differentiation as FAs are crucial for the production of many myelin sheath components.

## CONFLICT OF INTEREST

The authors declare no conflict of interest.

## Supporting information


**Figure S1** FABP7 in adult OPCs shows post‐translational modifications. (a–c) Western Blot analysis of posttranslational modifications of the adult form of FABP7. P = postnatal day. M = month. (a) Glycosylation and phosphorylation. (b) Ubiquitination. Ubq = Ubiquitin. (c) Sumoylation. (d) Western blot analysis of the specificity of the FABP7 (BLBP) antibody in the spinal cord, olfactory bulb and cerebral cortex at P21 in WT and *Fabp7*KO mice.

**Figure S2** FABP7 is not required for OPC proliferation during developmental myelination. (a) Immunohistochemistry staining for SOX10, NG2, and KI67 (marker of proliferating OPCs) in mixed glia derived OPCs after *Fabp7* siRNA knockdown (Scale bar: 100 μm). (b,c) Quantification of the percentage of DAPI^+^ cells expressing SOX10 (b) and SOX10^+^ cells expressing KI67 (unpaired Student's *t*‐test, *p* = .0315; *n* = 3, mean ± *SEM*) (c). (d) Immunohistochemistry staining for SOX10 and KI67 in the ventral white matter of the developing spinal cord (Scale bar: 100 μm). (e) Quantification of the percentage of SOX10^+^KI67^+^ cells in the white matter of the spinal cord of WT and *Fabp7*KO mice at P7 and P14 (unpaired Student's *t*‐test, *p* = .5839 (P7) and *p* = 0.2022 (P14); *n* = 5–6, mean ± *SEM*).

**Figure S3** FABP7 is not required for OPC proliferation during remyelination. (a) Immunohistochemistry staining for SOX10 and KI67 (marker of proliferating OPCs) in the lesion at 14dpl (Scale bar: 100 μm). (b) Quantification of SOX10^+^ cells per area in the lesion of WT and *Fabp7*KO mice at 5dpl and 14dpl (Unpaired Student's *t*‐test; *p* = .4534 (14dpl); *n* = 2 for 5dpl and *n* = 4 for 14dpl, mean ± *SEM*). (c) Quantification of the percentage of SOX10^+^KI67^+^ cells in the lesion of WT and *Fabp7*KO mice at 5dpl and 14dpl (unpaired Student's *t*‐test, *p* = .4534 (14dpl); *n* = 2 for 5dpl and *n* = 4 for 14dpl, mean ± *SEM*).

## Data Availability

The data supporting the findings of this study are available from the corresponding author upon request.
